# HIV Vpr Modulates the Host DNA Damage Response at Two Independent Steps to Damage DNA and Repress Double-Strand DNA Break Repair

**DOI:** 10.1128/mBio.00940-20

**Published:** 2020-08-04

**Authors:** Donna Li, Andrew Lopez, Carina Sandoval, Randilea Nichols Doyle, Oliver I. Fregoso

**Affiliations:** a Department of Microbiology, Immunology, and Molecular Genetics, University of California, Los Angeles, California, USA; b Molecular Biology Institute, University of California, Los Angeles, California, USA; University of Washington

**Keywords:** HIV, HIV-1, HIV-2, Vpr, DNA damage response, homologous recombination, DNA replication, host-pathogen interactions, RNA virus, DNA damage, DNA damage checkpoints, human immunodeficiency virus, lentiviruses, retroviruses, virus-host interactions

## Abstract

The DNA damage response (DDR) is a signaling cascade that is vital to ensuring the fidelity of the host genome in the presence of genotoxic stress. Growing evidence has emphasized the importance of both activation and repression of the host DDR by diverse DNA and RNA viruses. Previous work has shown that HIV-1 is also capable of engaging the host DDR, primarily through the conserved accessory protein Vpr. However, the extent of this engagement has remained unclear. Here, we show that HIV-1 and HIV-2 Vpr directly induce DNA damage and stall DNA replication, leading to the activation of several markers of double- and single-strand DNA breaks. Despite causing damage and activating the DDR, we found that Vpr represses the repair of double-strand breaks (DSB) by inhibiting homologous recombination (HR) and nonhomologous end joining (NHEJ). Mutational analyses of Vpr revealed that DNA damage and DDR activation are independent from repression of HR and Vpr-mediated cell cycle arrest. Moreover, we show that repression of HR does not require cell cycle arrest but instead may precede this long-standing enigmatic Vpr phenotype. Together, our data uncover that Vpr globally modulates the host DDR at at least two independent steps, offering novel insight into the primary functions of lentiviral Vpr and the roles of the DNA damage response in lentiviral replication.

## Abstract (precis)

The DNA damage response (DDR) is a signaling cascade that safeguards the genome from genotoxic agents, including human pathogens. However, the DDR has also been utilized by many pathogens, such as human immunodeficiency virus (HIV), to enhance infection. To properly treat HIV-positive individuals, we must understand how the virus usurps our own cellular processes. Here, we have found that an important yet poorly understood gene in HIV, Vpr, targets the DDR at two unique steps: it causes damage and activates DDR signaling, and it represses the ability of cells to repair this damage, which we hypothesize is central to the primary function of Vpr. In clarifying these important functions of Vpr, our work highlights the multiple ways human pathogens engage the DDR and further suggests that modulation of the DDR is a novel way to help in the fight against HIV.

## INTRODUCTION

Primate lentiviruses encode accessory proteins that enhance viral replication (1). This is achieved through direct interactions with host proteins to usurp their cellular functions or to antagonize their antiviral activity. HIV-1 encodes four accessory factors: Vpr, Vif, Vpu, and Nef. In addition, a subset of lentiviruses, including HIV-2, encode a paralog of Vpr, called Vpx (2). Of all the lentiviral accessory genes, *vpr* is the only gene with a still unknown primary function.

Despite this, Vpr is critical for the infectivity of HIV and related primate lentiviruses. *In vivo*, viruses lacking Vpr are attenuated compared to wild-type (WT) viruses, and the dominant viral species to emerge (i.e., most fit) have restored Vpr protein expression (3, 4). Furthermore, *vpr* is evolutionarily conserved by all extant primate lentiviruses (5). Together, this indicates that lentiviruses have maintained *vpr* for a highly important function. Of the many potential roles assigned to Vpr, activation of the host DNA damage response (DDR) and subsequent cell cycle arrest are the only phenotypes conserved by diverse Vpr orthologs (6–8). This conservation of function suggests that the engagement of the DDR is central to Vpr function.

The DDR is a protein signaling cascade that ensures the fidelity of the genome. It consists of sensors that recognize specific DNA lesions, mediators, and transducers, which transmit this signal of damaged DNA, and effectors, which directly execute a cellular response. Ataxia telangiectasia and Rad3 (ATR) (9), ataxia telangiectasia mutated (ATM) (10), and DNA-dependent protein kinase (DNA-PK) (11) are kinases at the head of the complex network that makes up the host DDR. The ATR kinase primarily responds to UV damage and replication stress, while ATM and DNA-PK participate in the repair of double-strand breaks (DSB) through homologous recombination (HR) and nonhomologous end joining (NHEJ), respectively (12). However, due to the essential role of the DDR, a tremendous amount of cross talk and redundancy exists between these kinases (13).

There is growing evidence that the DDR is important for viral replication, where it acts to both enhance and inhibit replication (14). For example, the DNA virus herpes simplex virus 1 (HSV-1) induces replication fork collapse at sites of oxidative damage (15). This leads to double-strand breaks (DSB), which initiate activation of the ATM repair pathway. HSV-1 infection also activates ATR, and the inactivation of either pathway severely compromises HSV-1 replication. RNA viruses also engage the DDR; for example, Rift Valley fever virus activates markers of DNA damage such as γH2AX and upregulates the ATM pathway but represses the ATR pathway (16). Contrary to enhancing viral replication, DDR proteins, such as DNA-PK (17), can activate an antiviral state upon sensing cytoplasmic DNA, while etoposide-induced DNA damage stimulates interferon via STING, ATM, and NF-κB (18–22). Together, these findings highlight the potential roles for the DDR in innate antiviral immunity and in enhancing viral replication.

Vpr engages the DDR at multiple steps. First, it causes G_2_ cell cycle arrest both *in vivo* and *in vitro* (7, 23–26). This arrest is dependent on ATR signaling, as it is blocked by the chemical inhibition of ATR (27). Moreover, Vpr-mediated cell cycle arrest requires interaction of Vpr with the Cul4A/DCAF1/DDB1 (CUL4A^DCAF1^) E3 ubiquitin ligase complex (28, 29), a cellular complex that is involved in many mechanisms of DNA repair (30, 31). Second, Vpr induces the expression, activation, and recruitment of DDR proteins, as assessed by immunofluorescence and Western blot analysis (32–34). Finally, in addition to the CUL4A^DCAF1^ ubiquitin ligase complex, Vpr interacts with and degrades many host DDR proteins, including UNG2 (35, 36), HLTF (37, 38), SLX4 complex proteins MUS81 and EME1 (34, 39), EXO1 (40), TET2 (41), MCM10 (42), and SAMHD1 (5, 43). Despite being one of the most highly conserved and robust phenotypes associated with Vpr, how Vpr engages the DDR at so many levels remains unclear.

Using a combination of DNA damage response assays, we monitored the induction of DNA damage, the early signaling events following DDR activation, and the cellular consequences associated with DNA damage and DDR activation. We found that Vpr engages the DNA damage response at two independent steps: it causes DNA damage and activates DDR signaling, and it represses double-strand DNA break repair. Using a panel of HIV-1 and HIV-2 Vpr mutants, we were able to separate these Vpr functions to show that while Vpr-induced DNA damage is independent of most known Vpr-host protein interactions, repression of double-strand break repair is dependent on DCAF1 recruitment. Finally, we showed that repression of HR repair is not a consequence of Vpr-mediated G_2_ cell cycle arrest, as it occurs prior to G_2_ arrest. Our data indicate that lentiviruses both activate and repress the DDR via Vpr and further characterize a novel phenotype of Vpr that can help explain many of the roles that have long been associated with Vpr.

## RESULTS

### HIV-1 and HIV-2 Vpr activate multiple DNA damage markers.

**FIG 1:**
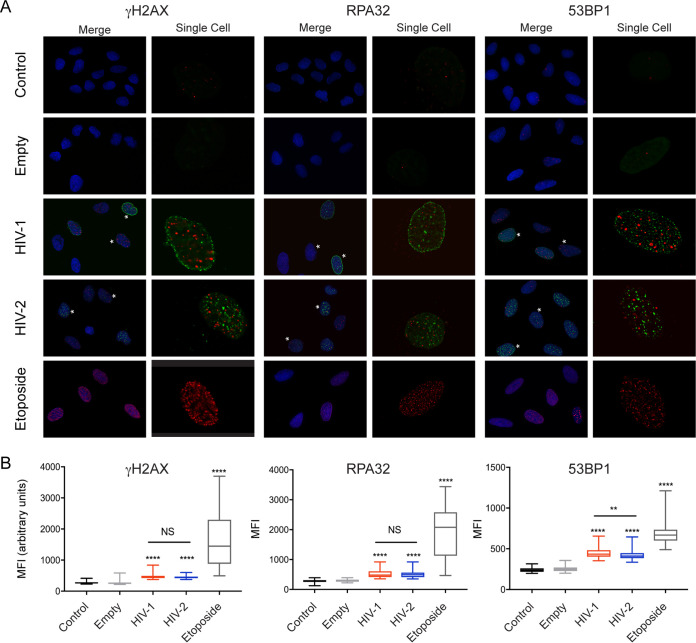
Activation of the DNA damage response is conserved between HIV-1 and HIV-2 Vpr. (A) Representative immunofluorescence images of U2OS cells infected with rAAV expressing 3× FLAG-tagged HIV-1 and HIV-2 Vpr, control empty vector (no Vpr), or uninfected control for 20 h. Blue (DAPI) shows the nuclei, 3×-FLAG Vpr is shown in green, and the phosphorylated DNA damage markers (γH2AX, RPA32, and 53BP1) are shown in red. Asterisks indicate cells with either high or low Vpr expression. The single-cell images show only 3×-FLAG Vpr and corresponding DNA damage marker. Images were taken at ×63 magnification. (B) Mean fluorescence intensity (MFI) of 100 cells per condition was quantified for all markers. Asterisks indicate statistical significance compared to empty vector control, as determined by Kruskal-Wallis tests (NS, nonsignificant; *, *P < *0.05; **, *P < *0.01; ***, *P* < 0.001; ****, *P < *0.0001; *n* = 2, one representative experiment shown).

The extent to which HIV Vpr engages the host DNA damage response (DDR) has not been critically examined. Therefore, we first asked if both HIV-1 and HIV-2 Vpr similarly activate the DDR. HIV-1 and HIV-2 are evolutionarily divergent primate lentiviruses that entered the human population through different nonhuman primate hosts (44). The Vpr proteins of these two viruses share only about 30 to 40% similarity, yet both cause cell cycle arrest (5, 7). Thus, if engagement of the DDR was central to the function of Vpr, we would expect that Vpr proteins from these two diverse human lentiviruses would also similarly activate the DDR. To test this, we delivered HIV-1 Q23-17 Vpr and HIV-2 Rod9 Vpr to U2OS cells via a recombinant adeno-associated virus (rAAV) vector system expressing 3× FLAG-tagged Vpr (6) and assayed for DDR markers 20 h postinfection by immunofluorescence (IF) for γH2AX, a marker for DNA double- and single-strand breaks (DSB and SSB, respectively) (45), RPA32, a marker of SSB (46), and 53BP1, a late marker of DSB that is recruited to sites of damage by γH2AX (47). In the presence of HIV-1 and HIV-2 Vpr, there were increased amounts of γH2AX foci compared to that of the uninfected and empty vector controls (Fig. 1), which correlated with G_2_ arrest (see Fig. S1A in the supplemental material). Similar to γH2AX, HIV-1 and HIV-2 Vpr expression also leads to increased levels of RPA32 and 53BP1 foci compared to those of uninfected and empty vector control cells and produced fewer, yet larger, foci than the etoposide positive control, a topoisomerase II inhibitor (Fig. 1). We also observed a distinct lack of colocalization between Vpr and markers of DNA damage (Fig. 1A), indicating that Vpr is not present at the potential sites of damage at this time point. Additionally, individual cells that expressed higher levels of Vpr did not have appreciably more DNA damage foci or higher mean fluorescence intensity (MFI) of γH2AX, RPA32, or 53BP1 per cell than cells with lower Vpr expression (Fig. 1A, asterisks, and Fig. S1B to E), suggesting that the activation of these markers is saturated with low levels of Vpr. While HIV-1 had significantly higher (*P* < 0.03) levels of 53BP1 than HIV-2 Vpr, the levels of γH2AX and RPA32 activation were the same for HIV-1 and HIV-2, as measured by MFI of individual cells (Fig. 1B).

10.1128/mBio.00940-20.1 FIG S1 HIV-1 and HIV-2 Vpr cause cell cycle arrest, and expression does not correlate with extent of DNA damage in U2OS cells (data are related to Fig. 1). (A) Representative bivariate cell cycle analysis flow cytometry plots for propidium iodide (PI; total DNA content) and EdU (DNA synthesis). Gating shows G_1_, S, and G_2_ populations of 10,000 U2OS cells treated under the same conditions as those for Fig. 1A (*n* = 4, one representative experiment shown). (B, C, and D) Mean fluorescence intensities (MFI) of individual cells for DAPI and indicated DNA damage response marker plotted against MFI of 3×-FLAG Vpr. Fifteen to 50 cells were measured per condition. Simple linear regression lines are shown. AU, arbitrary units. (E) *R*^2^ values from linear regression lines in panels B through D. Download FIG S1, TIF file, 1.7 MB. Copyright © 2020 Li et al. 2020 Li et al. This content is distributed under the terms of the Creative Commons Attribution 4.0 International license.

We also tested a number of HIV-1 and HIV-2 Vpr isolates to determine if activation of the DDR by HIV-1 and HIV-2 Vpr was isolate specific or conserved by the greater diversity of HIV Vpr proteins. These include representative Vpr isolates from HIV-1 group M (subtype G), N, O, and P consensus sequences, as well as HIV-2 Vpr isolates from groups A and B and divergent groups. We found that all HIV-1 and HIV-2 Vpr proteins tested caused cell cycle arrest and increased the number of γH2AX foci, indicative of DDR activation (Fig. S2). In total, our data highlight that a conserved function of HIV-1 and HIV-2 Vpr is the activation of the same markers of single- and double-strand DNA damage.

10.1128/mBio.00940-20.2 FIG S2 Cell cycle arrest and activation of DNA damage response are conserved by diverse HIV-1 and HIV-2 Vpr isolates (data are related to Fig. 1). (A) Representative univariate cell cycle analysis flow cytometry plots for uninfected control, empty vector, HIV-1 Q23-17, M.G SE6165 (a group M subtype G sequence), N consensus, O consensus, and P consensus and HIV-2 ROD9, 7312A (a group B sequence), A.PT (a group A sequence), and G.CI.92 (a divergent sequence) using PI staining. Gating shows G_1_ and G_2_ populations of 10,000 U2OS cells treated under conditions similar to those for Fig. 1A (*n* = 2, one representative experiment shown). (B) Box plot representation of the γH2AX MFI distribution for uninfected control, empty vector, HIV-1 Q23-17, M.G SE6165, N consensus, O consensus, and P consensus and HIV-2 ROD9, 7312A, A.PT, and GCI.92. One hundred cells were measured per condition and treated under conditions similar to those for Fig. 1A. Asterisks indicate statistical significance from empty vector control, as described for Fig. 1B (*n* = 2, one representative experiment shown). AU, arbitrary units. Download FIG S2, TIF file, 1.7 MB. Copyright © 2020 Li et al. 2020 Li et al. This content is distributed under the terms of the Creative Commons Attribution 4.0 International license.

### HIV-1 and HIV-2 Vpr expression damages DNA and induces replication stress.

**FIG 2:**
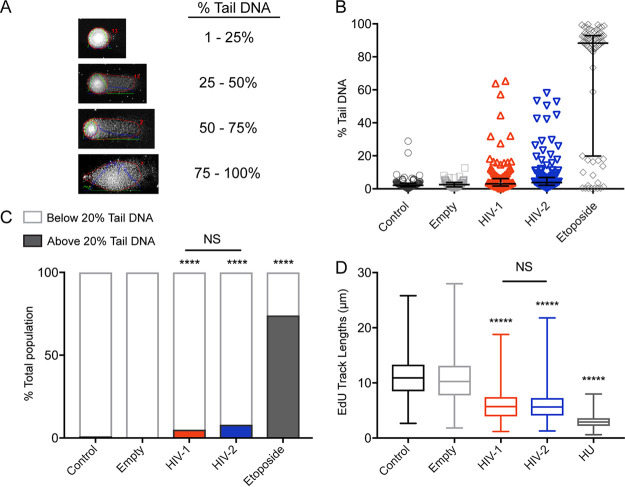
HIV-1 and HIV-2 Vpr damage DNA and stall DNA replication. (A) Visual representation taken from the alkaline comet assay of the four degrees of damage measured by percent tail DNA. Intensity profiles, lines, and numbers on the images were automatically generated by the *OpenComet* plug-in for the ImageJ software. (B) Distribution of the percent tail DNA measured for 100 cells per condition from one independent experiment using the *OpenComet* plug-in. U2OS cells were treated under the same conditions as those for Fig. 1A prior to being harvested for the comet assay. The bars represent the median with interquartile range; *n* = 3, one representative experiment shown. (C) A bar graph representation of the cells in panel B separated into two populations, below 20% tail DNA (unshaded) and above 20% tail DNA (shaded). Asterisks indicate statistical significance compared to empty vector control or HIV-1 compared to HIV-2 Vpr, as determined by chi-square test (NS, nonsignificant; *, *P < *0.05; **, *P < *0.01; ***, *P* < 0.001; ****, *P < *0.0001; *n* = 3, one representative experiment shown). (D) A box and whiskers representation of the distribution of EdU track lengths (μm). U2OS cells were treated under the same conditions as those shown in Fig. 1A. Asterisks indicate statistical significance for HIV-1, HIV-2, and hydroxyurea (HU) compared to the empty vector control, as determined by the Kruskall-Wallis test, while statistical difference between HIV-1 and HIV-2 was determined by the Mann-Whitney test (NS, nonsignificant; *, *P < *0.05; **, *P < *0.01; ***, *P* < 0.001; ****, *P < *0.0001; *n* = 3, one representative experiment shown).

The formation of γH2AX, RPA32, and 53BP1 foci in cells expressing HIV-1 and HIV-2 Vpr suggests the presence of both SSB and DSB. However, it is also possible that Vpr leads to activation of these markers without causing actual DNA damage. Previous studies to identify Vpr-induced DNA damage using pulsed-field gel electrophoresis, which only reveals DSB, have been contradictory (48, 49). Here, we used the alkaline comet assay, which uses a high-pH (>10) buffer to denature supercoiled DNA and single-cell gel electrophoresis to reveal damaged DNA fragments, including both SSB and DSB (50). U2OS cells were infected with rAAV-Vpr for 20 h, and the extent of DNA damage within individual cells was measured by calculating the percent tail DNA, which is proportionate to the amount of damaged DNA within a cell (Fig. 2A). While uninfected and empty vector control cells had little appreciable damage, both HIV-1 and HIV-2 Vpr expression significantly increased levels of percent tail DNA, indicative of an increase in damaged DNA (Fig. 2B). These results also correlate well with the IF data for γH2AX, RPA32, and 53BP1, which show lower MFI for Vpr-induced DNA damage markers than etoposide treatment (Fig. 1B). We segregated the samples into two populations, below and above 20% tail DNA, to highlight the population of cells within each sample with a greater extent of damage (Fig. 2A and C). Whereas approximately 1% of uninfected and empty vector control cells had tail DNA above 20%, HIV-1 and HIV-2 Vpr expression resulted in 5% and 8% of cells above 20% tail DNA, respectively, indicating that the expression of Vpr leads to significant DNA damage.

As replication stress has been proposed to be a driver of this Vpr-induced DDR (51) and the activation of the DNA damage markers and cell cycle arrest (Fig. 1A and Fig. S1) are hallmarks of stalled DNA replication forks, we next determined whether Vpr expression leads to replication fork stalling via the DNA combing assay (52). This assay quantitates the length of replication tracks by incorporation of EdU (5-ethynyl-2′-deoxyuridine) into nascent DNA. U2OS cells were infected with rAAV-Vpr for 20 h, at which point EdU was added to the cells for 20 min. Hydroxyurea (HU), which stalls DNA replication by depleting deoxynucleoside triphosphate pools (53), was used as a positive control. We found that HIV-1 and HIV-2 Vpr significantly decreased EdU track lengths compared to those of the uninfected and empty vector controls (Fig. 2D). Consistent with DNA damage markers, there was no direct correlation between levels of Vpr expression and DNA replication during this 20-min window. However, cells expressing the highest levels of Vpr were largely not in S-phase during this window (Fig. S3), suggesting there is a threshold where Vpr expression robustly excludes cells from S phase. Like the comet and IF assays, the greatest amount of replication fork stalling was exhibited by the positive control, HU, suggesting that while the impairment of normal DNA replication by HIV-1 and HIV-2 Vpr is significant, it is not as detrimental to the cell as HU. Overall, our alkaline comet and DNA combing data show that Vpr directly engages the DDR by inducing DNA breaks and stalling DNA replication.

10.1128/mBio.00940-20.3 FIG S3 DNA replication stalling does not correlate with levels of Vpr expression (data are related to Fig. 2). (A) Representative DNA replication analysis flow cytometry plots for EdU (DNA synthesis) and Hoechst (total DNA content). Gating shows S phase populations of 10,000 U2OS cells treated under the same conditions as those for Fig. 1A (*n* = 3, one representative experiment shown). (B) Contour plots of U2OS cells from S phase gate in panel A, plotted for mCherry and EdU to assay correlation between DNA replication and Vpr expression. (C) Mean fluorescence intensities (MFI) for mCherry and EdU from S phase gates in panel A. Download FIG S3, TIF file, 1.1 MB. Copyright © 2020 Li et al. 2020 Li et al. This content is distributed under the terms of the Creative Commons Attribution 4.0 International license.

### ATR senses stalled replication forks downstream of Vpr-induced DNA damage.

Our results indicate that Vpr directly damages DNA and stalls DNA replication (Fig. 2). However, whether DNA damage occurs prior to replication fork stalling or as a consequence of stalled replication forks is unclear. To differentiate between these two possibilities, we inhibited the fundamental DNA damage repair kinase ATR via the selective ATR inhibitor (ATRi) VE-821 (54). ATR acts as the primary signaling axis for replication stress and cell cycle checkpoints, where it is recruited during S phase through replication protein A (RPA) to stalled replication forks (9, 54). Here, it stabilizes replication forks from collapse, initiates the recruitment of repair proteins, and activates critical cell cycle checkpoints (9, 54). If Vpr-mediated DNA damage is due to stalled replication, we would expect ATR inhibition to increase DNA damage, as the cells would not be able to guard against replication fork collapse or initiate repair. However, if damage occurs before replication stress, we would expect the inhibition of ATR to alter fork progression but not DNA damage.

**FIG 3:**
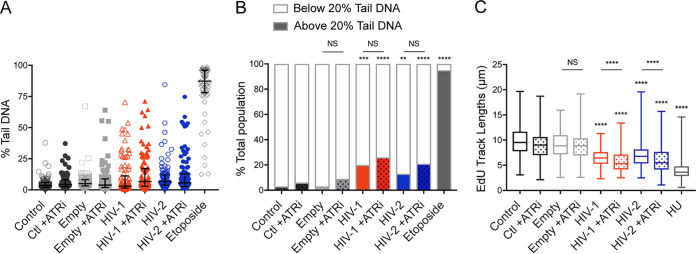
Vpr-induced DNA damage occurs prior to replication fork stalling and is independent of ATR. (A) U2OS cells treated under the same conditions as those for Fig. 1A were incubated with or without 10 μM VE-821 ATR inhibitor (ATRi) for 20 h and then subjected to the alkaline comet assay as described for Fig. 2B. Graph shows quantification of percent tail DNA of 100 cells measured per condition, with the bars representing the medians and interquartile ranges. ATRi-treated conditions are shown in filled shapes (*n* = 3, one representative experiment shown). (B) A bar graph representation of the data from panel A, with the population separated as shown in Fig. 2C. Cells treated with ATRi above 20% tail DNA are represented as the shaded regions with dots. Asterisks indicate statistical significance as determined by chi-square test (NS, nonsignificant; *, *P < *0.05; **, *P < *0.01; ***, *P* < 0.001; ****, *P < *0.0001; *n* = 3, one representative experiment shown). (C) Distribution of EdU track lengths (μm) from cells treated under the same conditions as those for panel A. Cells treated with ATRi are represented as box plots with dots. Asterisks indicate statistical significance of empty vector with ATRi, HIV-1 with or without ATRi, HIV-2 with or without ATRi, and etoposide compared to empty vector without ATRi, as determined by the Kruskall-Wallis test, while statistical difference between empty vector, HIV-1, and HIV-2 with or without ATRi was determined by the Mann-Whitney test (NS, nonsignificant; *, *P < *0.05; **, *P < *0.01; ***, *P* < 0.001; ****, *P < *0.0001; *n* = 3, one representative experiment shown).

We first confirmed ATR inhibition mitigated Vpr-mediated cell cycle arrest for both HIV-1 and HIV-2 Vpr isolates tested (Fig. S4A). We also assayed for an effect of ATM inhibition (ATMi; KU-55933), as we found activation of repair markers associated with ATM activation (such as γH2AX and 53BP1 in Fig. 1) but found no effect of ATMi on Vpr-mediated cell cycle arrest (Fig. S4B), consistent with previously published results (32, 49, 55). Next, to determine the effect of ATR inhibition on DNA damage by Vpr, we again used the alkaline comet assay. While all samples had proportionately increased levels of damage when ATR was inhibited, there was no significant difference for either HIV-1 or HIV-2 Vpr with or without ATRi (Fig. 3A and B). This suggests that ATR inhibition does not affect the ability of Vpr to generate DNA lesions.

10.1128/mBio.00940-20.4 FIG S4 ATR inhibition, but not ATM inhibition, blocks Vpr-mediated cell cycle arrest (data are related to Fig. 3). (A) Representative univariate cell cycle analysis flow cytometry plots for uninfected control, empty vector, HIV-1, HIV-2, and etoposide with or without the ATR inhibitor VE-821 (ATRi) under conditions similar to those for Fig. 3A, treated for 38 h and stained with PI. Gating shows G_1_ and G_2_ populations for 10,000 U2OS cells. (B) Representative univariate cell cycle analysis flow cytometry plots for uninfected control, empty vector, HIV-1, HIV-2, and etoposide with the ATM inhibitor KU-55933 (ATMi), as for panel A. Dimethyl sulfoxide (DMSO) was used as a control for ATRi and ATMi. Download FIG S4, TIF file, 2.1 MB. Copyright © 2020 Li et al. 2020 Li et al. This content is distributed under the terms of the Creative Commons Attribution 4.0 International license.

In contrast, the DNA combing assay, which we used to determine the effect of ATR inhibition on stalled replication fork progression by Vpr, showed that replication track lengths were significantly shorter for HIV-1 and HIV-2 Vpr-expressing cells when ATR was inhibited (Fig. 3C), presumably due to fork collapse. Although the overall effects of ATRi are modest, which is likely due to the intertwined nature of DNA damage, sensing, and repair, our data from the comet and DNA combing assays show that while Vpr-mediated DNA damage is independent of ATR signaling, the ability to stall DNA replication is not. Moreover, it indicates that Vpr first induces DNA damage, which leads to the activation of ATR and subsequent stalled replication forks, presumably to mitigate replication stress.

### Vpr sensitizes cells to additional double-strand breaks.

As we established with the immunofluorescence and alkaline comet assay, HIV-1 and HIV-2 Vpr induce DNA damage-activating markers related to a wide variety of DNA lesions, such as SSB and DSB. While our data suggest that Vpr directly damages DNA, it is also possible that damage results from the inability of cells to repair preexisting damage, such as damage due to replication stress. To address this question, we tested the sensitivity of cells expressing Vpr against various chemotherapeutics that directly damage DNA or inhibit a repair mechanism to cause damage.

**FIG 4:**
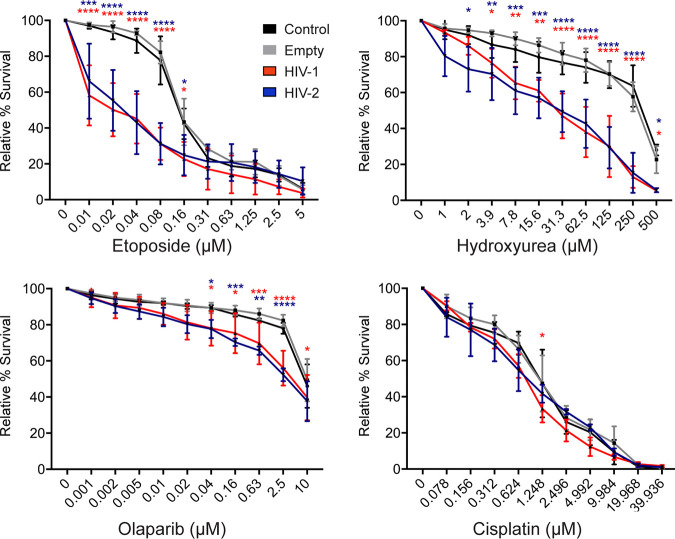
Cells expressing HIV-1 or HIV-2 Vpr are hypersensitive to exogenous double-strand DNA breaks. Sensitivities of the untreated control, empty vector control, HIV-1, and HIV-2 Vpr-expressing U2OS cells to etoposide, hydroxyurea, olaparib, and cisplatin were tested by incubating cells for 7 days in the corresponding drug at the indicated concentrations. Survival was analyzed by crystal violet staining for live cells compared to the no drug treatment. Sensitivity results are the means from three independent experiments (*n* = 3), and error bars represent ± standard deviations. Asterisks indicate statistical significance compared to empty vector control, as determined by 2-way analysis of variance (*, *P* < 0.05; **, *P* < 0.03;***, *P* < 0.002; ****, *P* < 0.0001).

We began by testing the sensitivity of Vpr-treated cells to etoposide, which generates DSB by preventing the enzyme topoisomerase II from properly removing knots formed from DNA overwinding (56). Cells expressing Vpr were highly sensitized to etoposide treatment, where survival at even the lowest concentration (0.01 μM) decreased to 60 to 70% compared to that of uninfected and empty vector control cells (Fig. 4). This indicates that Vpr-expressing cells are unable to repair etoposide-induced DSB. We next tested sensitivity to HU. Prolonged exposure of cells to HU at high concentrations results in replication fork collapse and extensive DSB (57). Although Vpr-expressing cells were not sensitized to HU treatment at low concentrations, at higher concentrations of HU (>3.90 μM), where DSB are presumably present, survival of cells expressing HIV-1 and HIV-2 was significantly decreased compared to that of control cells (Fig. 4). Similar results were seen for the PARP1/2 inhibitor olaparib, which also leads to DSB due to the inability to repair DNA lesions (58) (Fig. 4). In contrast to the other chemotherapeutics, HIV-1 and HIV-2 Vpr expression did not dramatically hypersensitize cells to the interstrand cross-linking agent cisplatin (59) (Fig. 4), despite activating markers associated with interstrand cross-link (ICL) repair (6, 34). Altogether, the sensitivity assays indicate that Vpr-expressing cells specifically show increased sensitivity to multiple chemotherapeutics that are capable of generating DSB by inhibiting crucial host repair mechanisms, suggesting that Vpr also inhibits the ability of cells to repair this damage.

### Vpr inhibits double-strand break repair.

**FIG 5:**
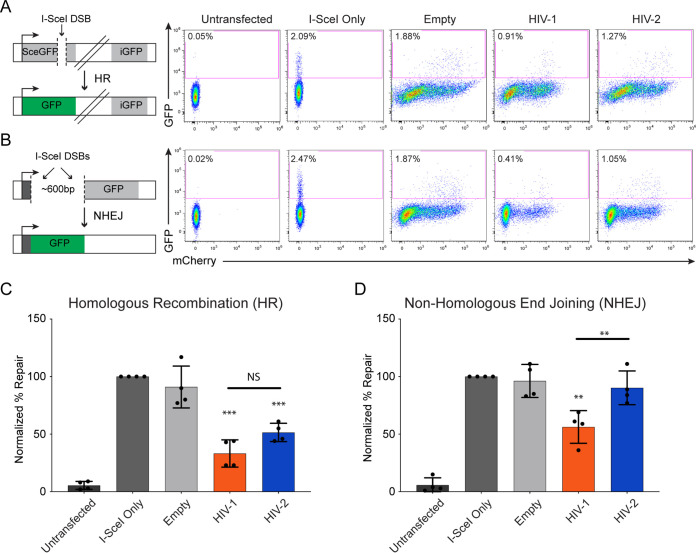
HIV-1 and HIV-2 Vpr repress double-strand break repair. (A, left) Schematic of I-SceI-based homologous recombination (HR) U2OS reporter cell line (DR-GFP assay). (Right) Representative flow cytometry plots of one I-SceI repair assay experiment for HR repair. Cells were transfected for 30 h with the I-SceI plasmid alone or with either empty vector, HIV-1, or HIV-2 Vpr that expresses mCherry via a T2A ribosomal skipping sequence. Twenty thousand cells were measured per condition and gated for homologous recombination-mediated DSB repair (GFP). (B, left) Schematic of I-SceI-based classical NHEJ U2OS reporter cell line (EJ5-GFP assay). (Right) Representative flow cytometry plots of one I-SceI repair assay experiment for NHEJ repair. Cells were treated and measured under the same conditions as those described for panel A. (C) I-SceI HR repair assay representing the average percent repair by homologous recombination from four experiments (*n* = 4), normalized to the I-SceI-only condition. Cells were treated and measured using the same conditions as those described for panel A. Asterisks indicate statistical significance compared to empty vector control, as determined by a one-sample *t* test (theoretical mean set to the average value of the empty vector control), while statistical difference between HIV-1 and HIV-2 was determined by the Mann-Whitney test (NS, nonsignificant; *, *P < *0.05; **, *P < *0.01; ***, *P* < 0.001; ****, *P < *0.0001). Error bars represent ± standard deviations. (D) I-SceI NHEJ repair assay representing average percent repair by classical nonhomologous end joining from four experiments (*n* = 4), normalized to the I-SceI-only condition. Cells were treated and measured under the same conditions as those described for panel A. Statistical analysis was determined with the same methods as those shown for panel C. Error bars represent ± standard deviations.

Because we observed that Vpr-expressing cells display hypersensitivity to the induction of exogenous DSB, we hypothesized that Vpr itself inhibits DNA break repair. To test this hypothesis, we used multiple independent green fluorescent protein (GFP)-based U2OS reporter cell lines that specifically monitor the repair of an I-SceI-induced DSB by either homologous recombination (HR), nonhomologous end joining (NHEJ), alternative NHEJ (alt-NHEJ), or single-strand annealing (SSA) (60, 61). Each cell line contains a GFP gene that is uniquely disrupted by an I-SceI restriction site and does not express GFP, as well as a truncated GFP donor sequence. Upon transfection and expression of I-SceI, this site is cut, and only proper repair by the indicated pathway results in GFP expression (Fig. 5A and B depict a schematic of HR and NHEJ cell lines, respectively). In addition to transfecting I-SceI alone, we also used combinations that included empty vector, HIV-1, or HIV-2 Vpr that express mCherry via a T2A ribosomal skipping sequence. Thirty hours later, we measured repair on a per-cell basis using flow cytometry for successful repair (GFP) and transfection efficiency (mCherry) (Fig. 5A and B).

We first tested the I-SceI reporter cell line for HR. While transfection of I-SceI alone or with empty vector control resulted in similar amounts of HR, we found that cells transfected with HIV-1 and HIV-2 Vpr decreased HR efficiency by 66% and 49%, respectively, when normalized to control cells at 100% (Fig. 5A and C). This indicates that HIV-1 and HIV-2 Vpr repress HR. Based on these results, we next tested the I-SceI reporter cell line that measures NHEJ, which is often utilized by cells to repair DSBs when HR is repressed (62). Similar to HR, HIV-1 Vpr expression also decreased NHEJ efficiency by 51% compared to that of wild-type cells. In contrast to HIV-1, HIV-2 Vpr did not significantly decrease NHEJ, as these cells were able to repair via NHEJ at 90% of wild-type levels (Fig. 5B and D), highlighting potential mechanistic differences between HIV-1 and HIV-2 Vpr. Consistent with DNA damage and DNA replication, there was no correlation between Vpr expression (mCherry) and repair (GFP) based on flow plots (Fig. 5A and B). Finally, we tested the I-SceI reporter cell lines for alt-NHEJ and SSA repair mechanisms but found no significant change in repair compared to control cells (Fig. S5). Thus, based on the data from the four different I-SceI reporter cell lines, we have identified that both HIV-1 and HIV-2 Vpr repress double-strand break repair, in addition to inducing DNA damage.

10.1128/mBio.00940-20.5 FIG S5 HIV-1 and HIV-2 Vpr do not alter other DNA repair pathways (data are related to Fig. 5). (A) I-SceI SSA assay representing average percent repair by single-strand annealing from three experiments (*n* = 3), normalized to the I-SceI-only condition. Cells were treated and analyzed as described for Fig. 5B. Error bars represent ± standard deviations. (B) I-SceI alt-NHEJ assay representing average percent repair by alternative nonhomologous end joining from three experiments (*n* = 3), normalized to the I-SceI-only condition. Cells were treated and analyzed as described for Fig. 5B. Error bars represent ± standard deviations. Download FIG S5, TIF file, 0.6 MB. Copyright © 2020 Li et al. 2020 Li et al. This content is distributed under the terms of the Creative Commons Attribution 4.0 International license.

### Disconnect between induction of DNA damage and downregulation of repair machinery.

Our findings demonstrate that both HIV-1 and HIV-2 Vpr are capable of inducing DNA damage, stalling DNA replication, downregulating double-strand DNA break repair, and causing cell cycle arrest. However, it is unclear how these phenotypes are linked and what role(s) host protein interactions play. To address these questions, we further tested a subset of well-characterized HIV-1 and HIV-2 Vpr mutants for their ability to induce, signal, and respond to DNA damage via the alkaline comet assay, EdU immunofluorescence, HR I-SceI repair assay, and bivariate cell cycle analysis, respectively. We tested four mutants for each HIV-1 and HIV-2 Vpr. These include HIV-1 W54R/HIV-2 L59A Vpr mutants, which block the ability of HIV-1 Vpr to recruit and degrade the DNA glycosylase UNG2 (63); HIV-1 Q65R/HIV-2 Q70R Vpr, which renders Vpr unable to properly localize, multimerize, or recruit known host proteins, such as the Cul4A^DCAF1^ complex or UNG2 and, therefore, is largely functionally dead (33, 64, 65); HIV-1 S79A/HIV-2 S84A mutants, which render Vpr unable to cause cell cycle arrest or interact with TAK1 to activate canonical NF-κB (66, 67); and HIV-1 R80A/HIV-2 R85A Vpr mutants, which can still interact with Cul4A^DCAF1^ and degrade TET2 (41) but do not cause cell cycle arrest, presumably due to the requirement of an additional unknown host protein(s) (68). Moreover, as HIV-1 and HIV-2 Vpr differentially interact with and/or downregulate UNG2, HLTF, and the SLX4 complex (6, 37), by testing diverse Vpr orthologs we were further able to dissect the requirement(s) for previously reported Vpr-interacting proteins in inducing DNA damage, stalling DNA replication, downregulating HR repair, and causing cell cycle arrest.

**FIG 6:**
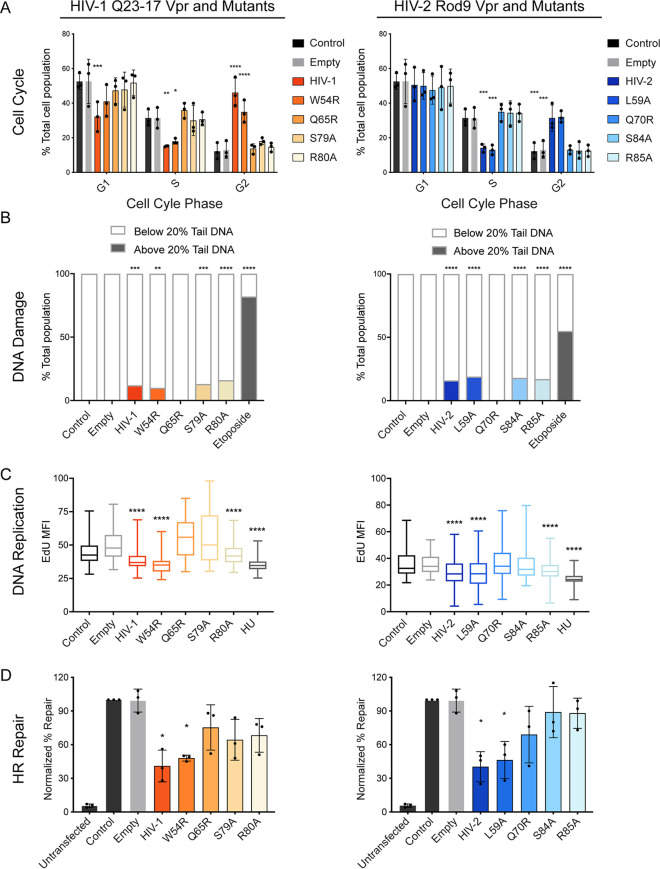
Vpr-induced DNA damage is independent of repression of homologous recombination and cell cycle arrest. (A) Bivariate cell cycle analysis of synchronized U2OS cells infected with rAAV expressing 3× FLAG-tagged HIV-1 and HIV-2 Vpr, empty vector, or control uninfected cells for 38 h. The graph shows the percentage of the population of 10,000 cells per condition in G_1_, S, and G_2_, measured using flow cytometry of cells stained for propidium iodide (PI; total DNA content) and EdU (DNA synthesis). Asterisks indicate statistical significance compared to empty vector control, as determined by Tukey’s multiple-comparison test (NS, nonsignificant; *, *P < *0.05; **, *P < *0.01; ***, *P* < 0.001; ****, *P < *0.0001; *n* = 3). Error bars represent ± standard deviations. (B) The alkaline comet assay for HIV-1 and HIV-2 Vpr mutants as represented in Fig. 2C with 100 cells measured per condition. U2OS cells were treated under conditions similar to those for Fig. 1A. Asterisks indicate statistical significance to empty vector control, as described for Fig. 2C (*n* = 3, one representative image shown). (C) Box and whisker plot representation of the distribution of EdU mean fluorescence intensity (MFI) for HIV-1 and HIV-2 Vpr mutants with cells treated under the same conditions as those for panel B. Asterisks indicate statistical significance to empty vector control, as determined by the Dunn’s multiple-comparison test (NS, nonsignificant; *, *P < *0.05; **, *P < *0.01; ***, *P* < 0.001; ****, *P < *0.0001; *n* = 3, one representative experiment shown). (D) Experimental results from the I-SceI DR-GFP assay representing average percent repair by homologous recombination for HIV-1 and HIV-2 mutants, as described for Fig. 5C. Asterisks indicate statistical significance from empty vector control, as described for Fig. 5C (*n* = 3).

10.1128/mBio.00940-20.6 FIG S6 HIV-1 and HIV-2 Vpr mutant analysis (data are related to Fig. 6). (A) Mutant Vpr protein expression. (Top) Anti-3× FLAG Vpr. (Bottom) Anti-actin as a control for equal total protein loading. (B) Representative bivariate cell cycle analysis flow cytometry plots for propidium iodide (PI; total DNA content) and EdU (DNA synthesis). Gating shows G_1_, S, and G_2_ populations of 10,000 U2OS cells treated under the same conditions as those for Fig. 1A (*n* = 3, one representative experiment shown), corresponding to Fig. 6A. (C) Distribution of the percent tail DNA measured for 100 cells per condition from the experiment shown in Fig. 6B using the *OpenComet* plug-in. Cells were treated and analyzed as described for Fig. 2B and 6B. Download FIG S6, TIF file, 2.3 MB. Copyright © 2020 Li et al. 2020 Li et al. This content is distributed under the terms of the Creative Commons Attribution 4.0 International license.

Consistent with previously published results, all mutants except HIV-1 W54R/HIV-2 L59A Vpr failed to induce cell cycle arrest (Fig. 6A and Fig. S6B). In contrast to cell cycle arrest, only HIV-1 Q65R/HIV-2 Q70R Vpr lost the ability to damage DNA (Fig. 6B and Fig. S6C), indicating that damage of DNA occurs independently of cell cycle arrest and of the Vpr-host protein-protein interactions assayed here. When testing for the effects of Vpr on DNA replication, we found that, in addition to HIV-1 Q65R/HIV-2 Q70R Vpr, HIV-1 S79A/HIV-2 S84A Vpr mutants were unable to stall DNA replication (Fig. 6C), suggesting that activation of TAK1 is integral in the ability of Vpr to stall DNA replication. Finally, in concert with cell cycle arrest, all mutants except the HIV-1 W54R/HIV-2 L59A Vpr mutants failed to repress homologous recombination repair (Fig. 6D). A summary of these results can be found in Table 1. Overall, our mutational analyses of HIV-1 and HIV-2 Vpr indicate that repression of HR and cell cycle arrest are correlated, and that these two phenotypes are independent of Vpr-induced DNA damage and downstream signaling. Moreover, by testing multiple mutants deficient for host factor recruitment, as well as comparing HIV-1 and HIV-2 Vpr orthologs, which differentially recruit host proteins, our results rule out most previously observed Vpr-interacting host proteins for a role in induction of DNA damage and repression of HR.

**TABLE 1:** Summary of mutant HIV-1 and HIV-2 Vpr data^*a*^

HIV	DNA damage	Replication stalling	HR repression	Cell cycle arrest
HIV-1/HIV-2	+	+	+	+
W54R/L59A	+	+	+	+
Q65R/Q70R	−	−	−	−
S79A/S84A	+	−	−	−
R80A/R85A	+	+	−	−

a Summary of data from Fig. 6. A plus sign indicates that Vpr was functional in the indicated assay, while a minus sign indicates that Vpr was statistically indistinguishable from the empty vector control.

### Repression of HR is not a consequence of Vpr-mediated cell cycle arrest.

The predominant phenotype of Vpr expression *in vivo* and *in vitro* is G_2_ cell cycle arrest. While it is unclear what leads to Vpr-mediated cell cycle arrest, G_2_ arrest depends on recruitment of the Cul4A^DCAF1^ ubiquitin ligase complex through a direct interaction of Vpr with DCAF1. Here, we have identified a new phenotype of Vpr, repression of HR, that tracks with G_2_ cell cycle arrest based on our Vpr mutant data (Fig. 6 and Table 1). However, whether repression of HR by Vpr is a consequence or potential driver of Vpr-mediated arrest remains unclear.

**FIG 7:**
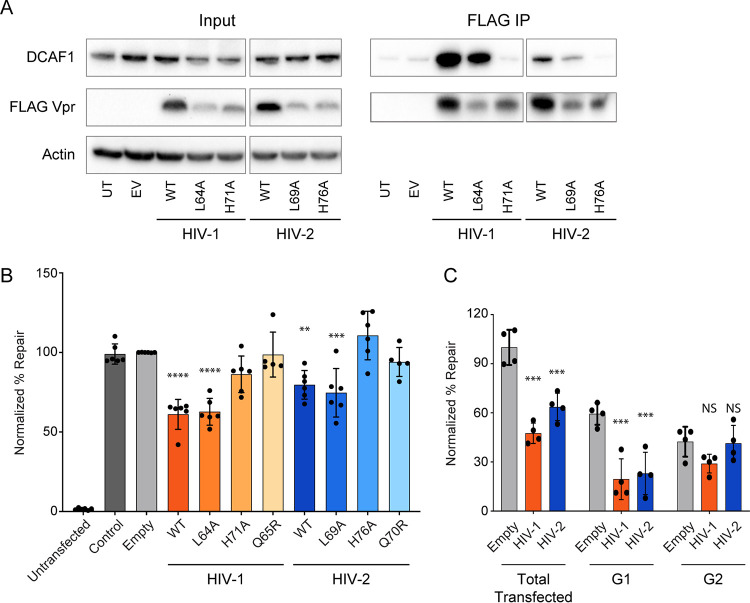
Repression of homologous recombination by Vpr requires DCAF1 but does not require cell cycle arrest. (A, left) Representative Western blots of U2OS cells for endogenous DCAF1, transiently transfected 3×-FLAG Vpr, and endogenous actin as a loading control. (Right) Immunoprecipitations against 3×-FLAG, probed for endogenous DCAF1 and transiently transfected 3×-FLAG Vpr. (B) Experimental results from the I-SceI DR-GFP assay representing average percent repair by homologous recombination for HIV-1 and HIV-2 mutants, as described for Fig. 5C. Asterisks indicate statistical significance from empty vector control, as described for Fig. 5C (*n* = 6). (C) Experimental results from bivariate I-SceI DR-GFP-cell cycle assay. Cells were transfected for 30 h with the I-SceI plasmid alone or with either empty vector, HIV-1, or HIV-2 Vpr that expresses mCherry via a T2A ribosomal skipping sequence and then labeled with Hoechst dye to label total DNA content. Twenty thousand cells were measured per condition. Total, G_1_, and G_2_ mCherry-expressing cell populations were gated for homologous recombination-mediated DSB repair (GFP). Asterisks indicate statistical significance from empty vector control, as described for Fig. 5C (*n* = 4).

To address this, we first asked if Cul4A^DCAF1^ complex recruitment is also required for repression of HR by Vpr. We selected two mutants that have been previously shown to alter HIV-1 Vpr binding to DCAF1, L64A (28) and H71R (35), and further generated those mutants in HIV-2 Vpr (L69A and H76R, respectively). To validate if these mutants lost the ability to recruit DCAF1, we immunoprecipitated FLAG-Vpr and probed for endogenous human DCAF1. In our hands, HIV-1 H71R/HIV-2 H76R no longer recruited DCAF1. However, HIV-1 L64A/HIV-2 L69A was still able to recruit the DCAF1 adaptor protein, though at a slightly lower level than wild-type Vpr (Fig. 7A). Consistent with recruitment of DCAF1, HIV-1 H71R/HIV-2 H76R, but not HIV-1 L64A/HIV-2 L69A, fully lost the ability to arrest cells (Fig. S7). We next tested these mutants for their ability to repress HR using the HR I-SceI repair assay. Again, consistent with DCAF1 binding and cell cycle arrest, HIV-1 H71R/HIV-2 H76R failed to repress HR, whereas HIV-1 L64A/HIV-2 L69A repressed HR to nearly WT Vpr levels (Fig. 7B). These data suggest that, similar to cell cycle arrest, repression of HR repair by Vpr requires DCAF1 binding.

10.1128/mBio.00940-20.7 FIG S7 HIV-1 and HIV-2 Vpr DCAF1-binding mutant analysis (data are related to Fig. 7). Representative univariate cell cycle analysis flow cytometry plots for uninfected control, empty vector, HIV-1, HIV-2, and mutants. Cells were treated for 38 h and stained with PI. Gating shows G_1_ and G_2_ populations for 10,000 U2OS cells. Download FIG S7, TIF file, 1.8 MB. Copyright © 2020 Li et al. 2020 Li et al. This content is distributed under the terms of the Creative Commons Attribution 4.0 International license.

To determine if repression of HR by Vpr requires G_2_ arrest or occurs independently of this arrest, we defined the cell cycle status (G_1_ or G_2_ phase) of DR-GFP cells that exhibited repair using Hoechst dye. Here, we first performed the DR-GFP assay as before. Cells were then stained with Hoechst dye to label DNA content, gated for Vpr expression (mCherry), and repair was measured for either total Vpr-expressing cells, Vpr-expressing cells in G_1_, or Vpr-expressing cells in G_2_. We would expect that if Vpr-mediated G_2_ arrest is required to repress HR, then Vpr-expressing cells in G_2_ would primarily show repressed HR when normalized to empty vector control cells. However, if G_2_ arrest is not required for Vpr to repress HR, then cells in G_1_ would also show a repression of HR in the presence of Vpr.

As seen previously, both HIV-1 and HIV-2 Vpr repressed total cellular HR, unlike the empty vector control. Vpr-expressing cells also showed strong repression of HR repair in G_1_ compared to that of empty vector control cells. However, Vpr-expressing cells did not repress HR in G_2_, as they were statistically indistinguishable from control cells (Fig. 7C). Together, these data indicate that Vpr-mediated repression of HR does not require G_2_ arrest but instead occurs primarily in the G_1_ phase of the cell cycle.

## DISCUSSION

Here, we show that HIV-1 and HIV-2 Vpr induce both double- and single-strand DNA breaks, leading to the recruitment of repair factors, including γH2AX, RPA32, and 53BP1. These Vpr-induced DNA lesions are sensed by ATR and require NF-κB signaling to stall DNA replication. However, contrary to the induction of DNA damage and the activation of the DNA damage response, Vpr represses essential mechanisms of double-strand break repair, including homologous recombination repair (HR) and nonhomologous end joining (NHEJ). Mutational analysis of Vpr has identified that there is a disconnect between mutants that can damage DNA and those that can repress DNA repair and activate cell cycle arrest. Finally, we show that repression of HR is not a consequence of G_2_ cell cycle arrest. Overall, our data support a model where Vpr has two unique and independent mechanisms to modulate the host DDR. First, Vpr has the inherent ability to induce DNA damage, which is largely independent of known Vpr-binding host factors. This Vpr-induced damage is sensed by ATR and signals through NF-κB to block DNA replication fork progression. Second, through recruitment of the Cul4A^DCAF1^ complex, Vpr represses DNA double-strand break repair machinery, leading to a prolonged cell cycle to deal with the inability to repair DNA lesions.

Why would Vpr engage the DDR at two unique steps, and how would this help lentiviral replication? While it may seem counterintuitive to both activate and repress the DDR through unique mechanisms, Vpr is not the only viral protein, and lentiviruses are not the only viruses, to both activate and repress the DDR at different steps in viral replication (14). For example, human papillomaviruses (HPV) upregulate ATM to push cells away from NHEJ and toward HR, which is thought to enhance viral persistence and integration (69, 70). Interestingly, this also sensitizes HPV^+^ cells to exogenous genotoxic agents due to their inability to repair additional damage (71), as we have shown here for HIV Vpr (Fig. 4). Moreover, as Vpr has two unique phases in an infected cell, i.e., it is delivered early via the incoming virion and expressed *de novo* following integration and gene expression, it is possible that these two distinct DDR-associated functions of Vpr are separated in the viral life cycle of an infected cell.

While it is possible that some of these DDR-associated phenotypes are indirect consequences of other effects of Vpr on the cell, such as induction of proinflammatory cytokines (72), this dual function of Vpr in engaging the DDR at multiple independent steps could help clarify some of the discrepancies in the Vpr literature and may directly explain many of the roles in viral replication attributed to Vpr (73–79). For example, DNA damage promotes nucleotide biosynthesis (80) and, thus, may enhance early events in HIV replication, such as reverse transcription. This is analogous to the degradation of SAMHD1 by lentiviral Vpx/Vpr (5, 81, 82) and could help to explain why Vpr from HIV-2, which encodes both Vpr and the paralogous Vpx protein, does not attenuate host repair machinery, or recruit host DDR proteins (6, 36, 37, 40, 41, 83), as efficiently as HIV-1 Vpr. The stalling of replication forks (Fig. 2D) could enhance integration by remodeling histones and prolonging the S phase. Integration could also be enhanced by attenuating double-strand break repair (Fig. 5), similar to the repression of HR and base excision repair by human T-lymphotropic virus 1 (HTLV-1), to facilitate viral integration (84–86). Moreover, the induction of DNA breaks (Fig. 2A to C) could enhance long terminal repeat-driven transcription by activating important DDR-responsive transcription factors, such as NF-κB and AP-1 (67, 87).

As the primary role of lentiviral accessory genes is to overcome antiviral restriction factors, our data also support a model where DDR proteins and/or pathways restrict HIV replication and are overcome by Vpr. This is consistent with the growing evidence that DDR proteins and pathways contribute to the innate immune response to pathogens (17–22). We have shown that, like Vpr-mediated cell cycle arrest, recruitment of the Cul4A ubiquitin ligase complex adaptor protein DCAF1 is required for repression of HR repair (Fig. 7). Vpr could be recruiting this complex away from a natural target or usurping it to degrade a host protein, which is consistent with the primary role of lentiviral accessory genes in viral replication, such as Vpx-mediated degradation of the antiviral DDR protein SAMHD1 (88, 89). While Vpr has been shown to recruit and degrade many host proteins, through the combination of our mutant data and use of HIV-1 and HIV-2 Vpr orthologs (Fig. 6 and 7), we are able to rule out most known DDR-associated Vpr-interacting proteins (and potential cellular effects of Vpr) for roles in modulating the DDR as described here. Whether some of the remaining Vpr-interacting proteins we were unable to characterize, such as the endonuclease Exo1, are required for Vpr-mediated engagement of the DDR or whether novel undiscovered host proteins are required remains unclear. Moreover, whether modulation of DDR pathways is a direct primary effect of Vpr or a consequence of degradation of an antiviral host protein that is also integral to the DDR is also unclear. However, our data pinpoint double-strand DNA break repairs as important cellular pathways that warrant further investigation into both innate immunity and Vpr.

Our mutant data also show that the long-standing enigmatic cell cycle arrest caused by Vpr correlates with repression of HR, suggesting these two phenotypes are linked. As HR is upregulated in G_2_, one might expect Vpr to enhance this repair mechanism instead of inhibit it. Intriguingly, we find the majority of Vpr-mediated repression of HR occurs in cells that are currently in G_1_, not G_2_, suggesting that repression of HR precedes, and may initiate, G_2_ arrest. Based on this, we hypothesize that repression of HR, not cell cycle arrest, is the crucial phenotype associated with Vpr, and that understanding this process will give clearer insight into the primary function of Vpr in viral replication.

Thus, while it is clear that the DDR is a central hub that is essential for replication of many viruses in different phases of their life cycle, the precise roles of Vpr-mediated activation and repression of the DDR in HIV replication remain obscure. In establishing that Vpr activates and represses the DDR, we have clarified the multiple ways that Vpr modulates the host DDR and uncovered a new phenotype for Vpr that may precede cell cycle arrest, suppression of double-strand break repair. This will allow us to better define the primary evolutionarily conserved role of Vpr. Finally, our data indicate that Vpr expression has important implications for the development and treatment of HIV-associated diseases such as cancer, where induction of DNA damage and deregulation of repair could serve to complicate tumorigenesis but also sensitize cells to chemotherapeutics, further highlighting the importance of Vpr in HIV replication and associated diseases.

## MATERIALS AND METHODS

### Plasmids.

pscAAV-mCherry-T2A-Vpr plasmids were generated by replacing GFP with mCherry from pscAAV-GFP-T2A-Vpr (6). HIV-2 A.PT (A.PT.x.ALI.AF082339) and HIV-2 G.CI.92 (G.CI.92.Abt96.AF208027) were synthesized as gBlocks (IDT) and subcloned into the pscAAV-mCherry-T2A-Vpr construct using standard cloning techniques. Vpr mutants were generated using site-directed mutagenesis (Q5 site-directed mutagenesis kit; NEB). pCBASceI was a gift from Maria Jasin (Addgene plasmid number 26477) (90).

### Cell lines and cell culture.

Human embryonic kidney (HEK) 293, HEK 293T, and human bone osteosarcoma epithelial (U2OS) cells were cultured as adherent cells directly on tissue culture plastic (Greiner) in Dulbecco’s modified Eagle’s medium (DMEM) growth medium (high glucose, l-glutamine, no sodium pyruvate; Gibco) with 10% fetal bovine serum (Gibco) and 1% penicillin-streptomycin (Gibco) at 37°C and 5% CO_2._ All cells were harvested using 0.05% trypsin-EDTA (Gibco). Transfections were performed with TransIT-LT1 (Mirus). The panel of U2OS cells containing an integrated reporter (DR-GFP, SA-GFP, EJ2-GFP, and EJ5-GFP) used in the I-SceI repair assays were kindly provided by Jeremy M. Stark (Beckman Research Institute of the City of Hope) (60).

### Generation of viruses.

AAV vectors were generated by transient transfection of HEK 293 cells using polyethyleneimine (PEI) as previously described (91). Levels of DNase-resistant vector genomes were quantified by inverted terminal repeat (ITR)-specific quantitative PCR (qPCR) using a linearized plasmid standard according to the method of Aurnhammer et al. (92).

### Western blots and coimmunoprecipitations.

Cells were lysed in radioimmunoprecipitation assay (RIPA) buffer (50 mM Tris-HCl [pH 8.0], 150 mM NaCl, 1 mM EDTA, 0.1% SDS, 1% NP-40, 0.5% sodium deoxycholate, Benzonase, protease inhibitor) and clarified by centrifugation at 14,500 × *g* for 10 min. Immunoprecipitations were performed as previously described (6) using anti-FLAG affinity beads (Sigma). All samples were boiled in 4× sample buffer (40% glycerol, 240 mM Tris, pH 6.8, 8% SDS, 0.5% β-mercaptoethanol, and bromophenol blue) in preparation for SDS-PAGE using 4 to 12% Bis-Tris polyacrylamide gels and subsequently transferred onto a polyvinylidene difluoride membrane. Immunoblotting was performed using mouse anti-FLAG M2 (Sigma), mouse anti-actin (Thermo-Fisher), rabbit anti-DCAF1 (Cell Signaling), goat anti-mouse horseradish peroxidase (HRP; Invitrogen), and goat anti-rabbit HRP (Invitrogen).

### DNA combing assay.

The DNA combing assay was adapted from reference 52. Cells were plated in 6-well tissue culture-treated plates (Greiner) at 1.75 × 10^6^ cells/well and allowed to rest overnight. Cells were then infected with rAAV 2.5 at equal titers (1.4 × 10^8^ copies/well) or 500 μM hydroxyurea (Sigma) for 20 h. Following infection, cells were incubated with 10 μM EdU (Invitrogen) for 20 min, harvested, spun down, and resuspended in 1× phosphate-buffered saline (PBS; Gibco). The cell suspension was added and lysed with lysis buffer (50 mM EDTA, 0.5% SDS, 200 mM Tris-HCl, pH 7.5) directly on a silane-coated slide (Electron Microscopy) and then incubated for 5 to 8 min. After incubation, the slide was tilted at a 45° angle to allow the droplet to roll down and then fixed with 3:1 methanol acetic acid for 15 min after the slide was completely dry. The slide then was washed with 1× PBS, blocked with 3% bovine serum albumin (BSA) for 30 min, and stained with secondary EdU mixture (Click-IT EdU imaging kit; Invitrogen) and DNA (Yoyo-1; Life Technologies). Microscopy was performed using the Zeiss Axio Imager Z1, and images were analyzed using ImageJ.

### Alkaline comet assay.

The alkaline comet assay was performed as previously described (50), with some minor changes. Cells were plated in 6-well tissue culture-treated plates (Greiner) at 1.75 × 10^6^ cells/well and allowed to rest overnight. Cells were then infected with rAAV 2.5 at equal titers (1.4 × 10^8^ copies/well) or 50 μM etoposide (Sigma) for 20 h. Following infection, cells were then harvested, spun down, and resuspended in 0.5% low-melting-point agarose at 37°C. Samples then were spread onto agarose-coated slides (Cell Biolabs) and allowed to solidify for 20 min at 4°C. After agarose solidification, samples were incubated in lysis buffer (10 mM Tris-HCl, pH 10, 2.5 M NaCl, 0.1 M EDTA, 1% Triton X-100) for 1 h and then in the alkaline running buffer (0.3 M NaOH, 1 mM EDTA) for 30 min and finally electrophoresed at 300 mA for 30 min, all done at 4°C. Samples then were washed in double-distilled water (ddH_2_O) and fixed in 70% ethanol at 4°C. Cells were stained with Yoyo-1 (Life Technologies) for 15 min at room temperature and then washed with ddH_2_O and dried overnight. Images were acquired on the Zeiss Axio Imager Z1. Images were analyzed using the OpenComet plug-in for ImageJ.

### Cell cycle analysis.

U2OS cells were plated and either left unsynchronized or synchronized using serum starvation with 0.05% fetal bovine serum (FBS)-DMEM (Gibco) for at least 12 h. Cells were infected with rAAV 2.5 (600 copies/cell) for 38 h. For labeling with Hoechst, cells were incubated with Hoechst ready flow reagent (Invitrogen) as recommended. For labeling with propidium iodide, cells were fixed with ice-cold ethanol, and DNA was stained with 0.01 g/ml propidium iodide (Sigma-Aldrich) and RNase A in PBS. For bivariate labeling, cells were additionally pulse labeled with 10 μM EdU (Invitrogen) for at least 30 min. Pulse-labeled cells were then permeabilized with 0.01% Triton X-100 for 3.5 min and fixed with 4% paraformaldehyde (PFA) for 20 min. EdU was detected using Click-iT EdU Alexa Fluor 647 imaging kit (Invitrogen) followed by Hoechst or PI staining. Cells were assessed by flow cytometry on a FACSVERSE (BD). At least 10,000 cells were collected each run, and data were analyzed using FlowJo software.

### Immunofluorescence.

Cells were plated in 6-well tissue culture-treated plates (Greiner) at 1.75 × 10^6^ cells/well and allowed to rest overnight. Cells were then infected with rAAV 2.5 at equal titers (1.4 × 10^8^ copies/well) or 50 μM etoposide (Sigma) for 20 h. For the EdU-IF experiments, EdU was added to the cells for 20 min. Cells were then permeabilized with 0.5% Triton X-100 in PBS at 4°C for 5 min and fixed in 4% PFA for 20 min. Samples were then washed in 1× PBS and incubated with blocking buffer (3% BSA, 0.05% Tween 20, and 0.04 NaN_3_ in PBS) for 30 min. Cells were probed with appropriate primary antibodies (anti-FLAG M2 [Sigma-Aldrich], anti-γH2AX, anti-RPA32 [GeneTex], or anti-53BP1 [Cell Signaling]) and then washed in PBST (0.05% Tween 20 in PBS) and probed with Alexa Fluor-conjugated secondary antibodies (Life Technologies). Nuclei were stained with diamidino-2-phenylindole (DAPI; Life Technologies). Secondary staining for EdU was added as the last step and stained twice to ensure signal. Images were acquired on the Zeiss Axio Imager Z1, and mean fluorescence intensity (MFI) was analyzed using ImageJ.

### Sensitivity assays.

Sensitivity assays were performed as previously described (93), with minor changes. Cells were plated in 24-well plates at 3 × 10^3^ cells/well and allowed to settle overnight. Done in triplicate per sample, the corresponding amounts of drugs were added and infected with rAAV 2.5 in equal titers (9.9 × 10^6^ copies/well) and then incubated for 7 days. On the 7th day, cells were washed with 1× PBS, fixed with 10% methanol and 10% acetic acid in water for 10 to 15 min, and stained with 0.1% crystal violet in methanol for 5 min. Plates were then washed with water and allowed to dry overnight, and the crystal violet was resolubilized with 300 μl 0.1% SDS in methanol for 2 h. A volume of 100 μl of the resolubilized dye was added to a 96-well, round-bottom plate (Greiner), and the absorbance was measured using a Gen5 (Biotek) plate reader at 595-nm wavelength.

### I-SceI repair assays.

I-SceI repair assays were performed as previously described (94), with some minor changes. Cells were plated in 6-well plates at 1.75 × 10^6^ cells/well and allowed to settle overnight. Cells were transfected with 1.5 μg pBASce-1 and 0.5 μg of corresponding pscAAV using Lipofectamine 3000 (Invitrogen) in antibiotic- and serum-free medium. Prior to transfection, cell medium was changed to DMEM high-glucose (Gibco) and l-glutamine (Gibco) and 5% fetal bovine serum (Gibco) without antibiotics. Cells were allowed to incubate with transfection reaction for 30 to 48 h, harvested, fixed with 4% PFA, and resuspended in fluorescence-activated cell sorting buffer (3% BSA in PBS). At least 20,000 cells/condition were measured through flow cytometry (Attune NxT), and data were analyzed using FlowJo.
