# Supplementary figures and images for: HIV Vpr Modulates the Host DNA Damage Response at Two Independent Steps to Damage DNA and Repress Double-Strand DNA Break Repair
Source: mBio. 2020 Aug 4;11(4):e00940-20. doi: 10.1128/mBio.00940-20 (PMC7407082; doi:10.1128/mBio.00940-20)

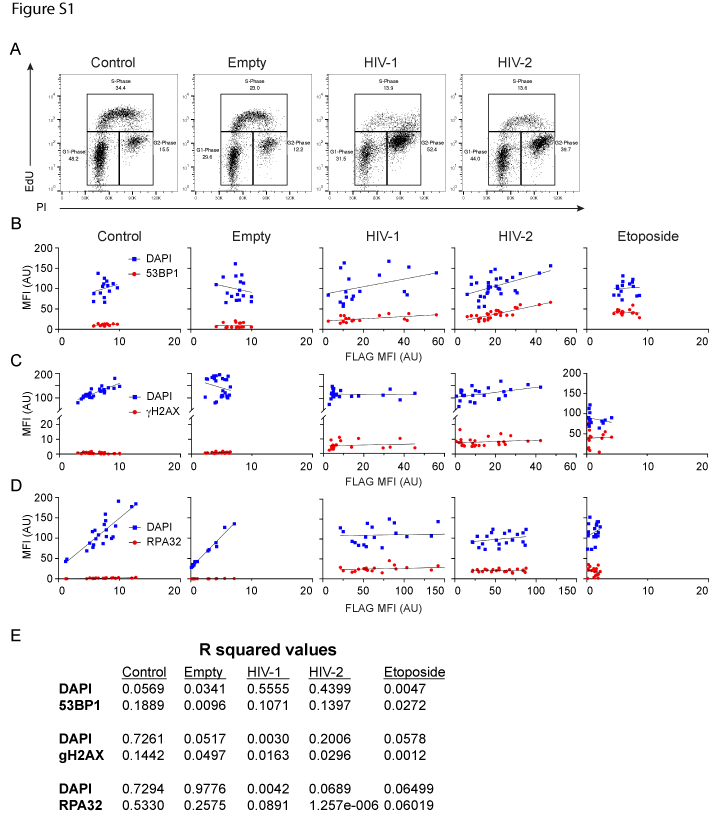

Supplement: FIG S1 [file mBio.00940-20-sf001.tif]

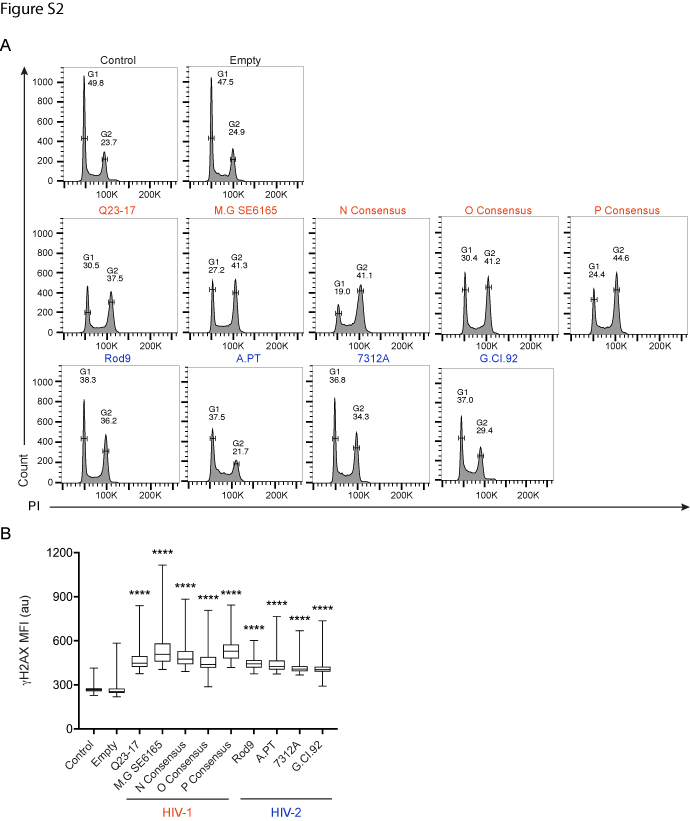

Supplement: FIG S2 [file mBio.00940-20-sf002.tif]

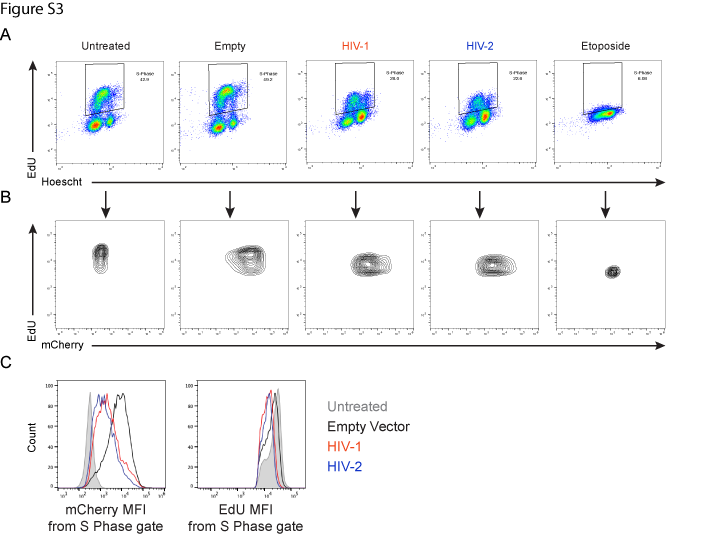

Supplement: FIG S3 [file mBio.00940-20-sf003.tif]

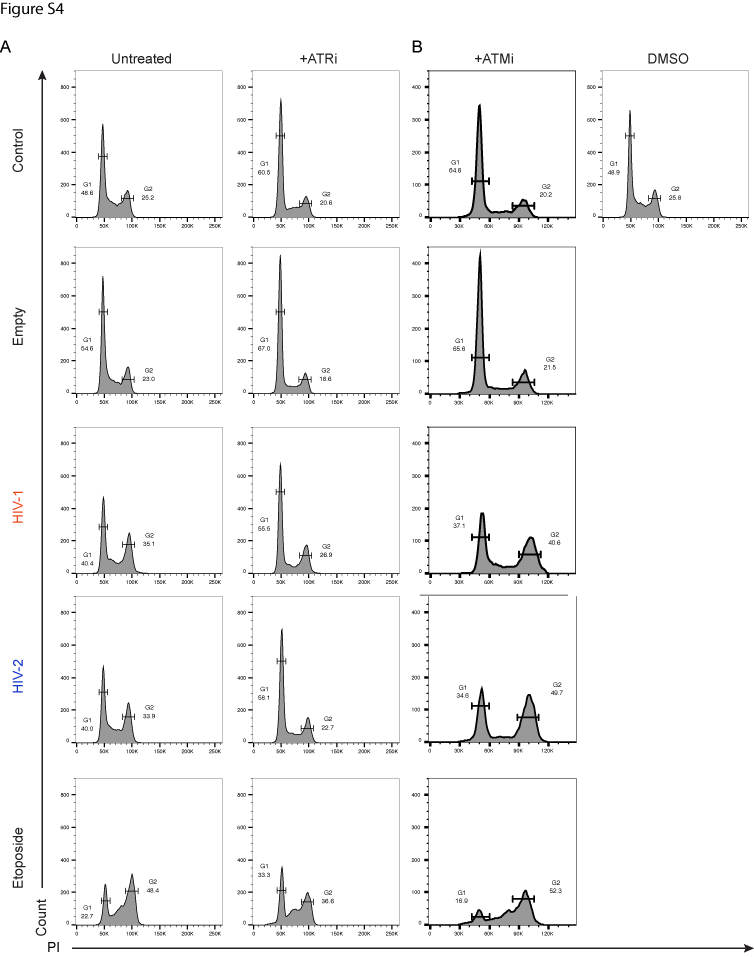

Supplement: FIG S4 [file mBio.00940-20-sf004.tif]

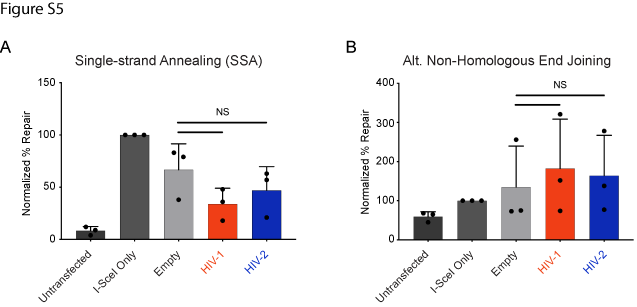

Supplement: FIG S5 [file mBio.00940-20-sf005.tif]

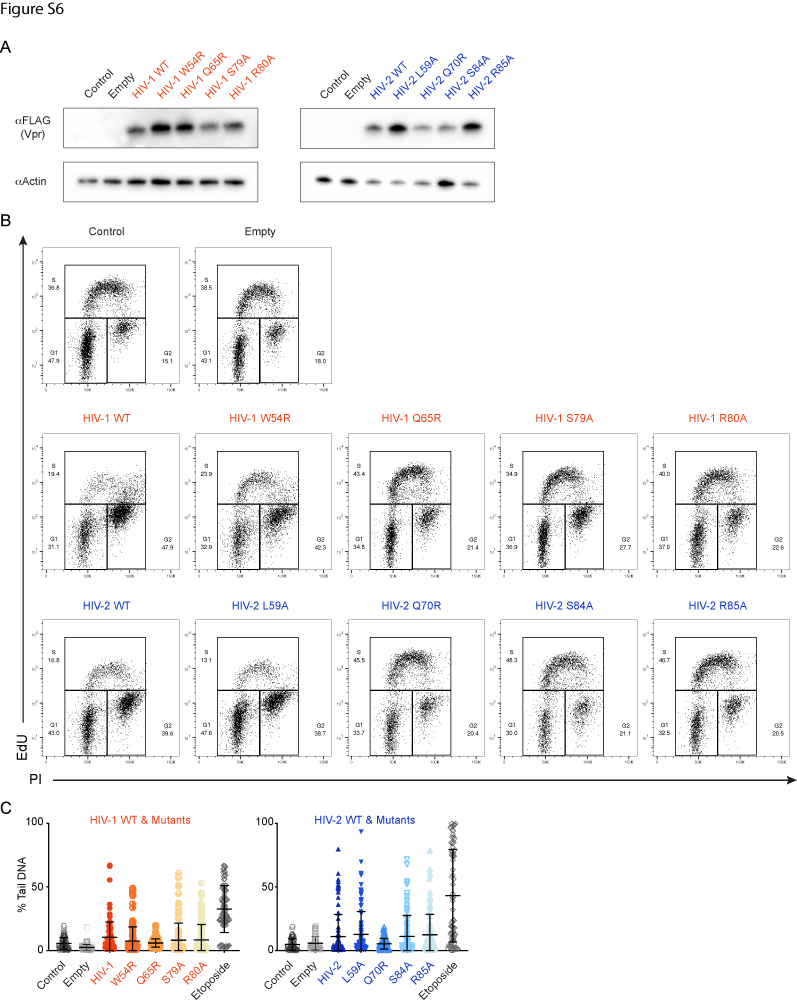

Supplement: FIG S6 [file mBio.00940-20-sf006.tif]

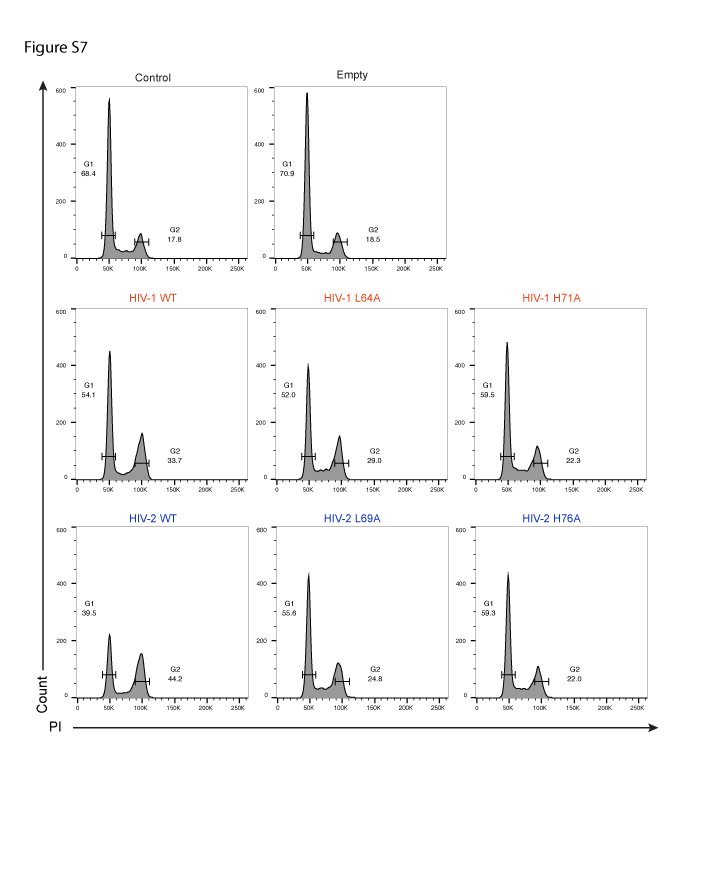

Supplement: FIG S7 [file mBio.00940-20-sf007.tif]
